# A specific super-enhancer actuated by berberine regulates EGFR-mediated RAS–RAF1–MEK1/2–ERK1/2 pathway to induce nasopharyngeal carcinoma autophagy

**DOI:** 10.1186/s11658-024-00607-4

**Published:** 2024-06-28

**Authors:** Yao Wu, Qunying Jia, Qi Tang, Lin Chen, Hongyu Deng, Yingchun He, Faqing Tang

**Affiliations:** 1 Hunan Key Laboratory of Oncotarget Gene and Clinical Laboratory, The Affiliated Cancer Hospital of Xiangya School of Medicine, Central South University and Hunan Cancer Hospital, Changsha, 410013 China; 2 The First Clinical College of Traditional Chinese Medicine of Hunan University of Chinese Medicine, and Hunan Cancer Hospital, Changsha, 410007 China

**Keywords:** BBR, EGFR, Autophagy, Super-enhancer, Nasopharyngeal carcinoma

## Abstract

Nasopharyngeal carcinoma (NPC), primarily found in the southern region of China, is a malignant tumor known for its highly metastatic characteristics. The high mortality rates caused by the distant metastasis and disease recurrence remain unsolved clinical problems. In clinic, the berberine (BBR) compound has widely been in NPC therapy to decrease metastasis and disease recurrence, and BBR was documented as a main component with multiple anti-NPC effects. However, the mechanism by which BBR inhibits the growth and metastasis of nasopharyngeal carcinoma remains elusive. Herein, we show that BBR effectively inhibits the growth, metastasis, and invasion of NPC via inducing a specific super enhancer (SE). From a mechanistic perspective, the RNA sequencing (RNA-seq) results suggest that the RAS–RAF1–MEK1/2–ERK1/2 signaling pathway, activated by the epidermal growth factor receptor (EGFR), plays a significant role in BBR-induced autophagy in NPC. Blockading of autophagy markedly attenuated the effect of BBR-mediated NPC cell growth and metastasis inhibition. Notably, BBR increased the expression of EGFR by transcription, and knockout of EGFR significantly inhibited BBR-induced microtubule associated protein 1 light chain 3 (LC3)-II increase and p62 inhibition, proposing that EGFR plays a pivotal role in BBR-induced autophagy in NPC. Chromatin immunoprecipitation sequencing (ChIP-seq) results found that a specific SE existed only in NPC cells treated with BBR. This SE knockdown markedly repressed the expression of EGFR and phosphorylated EGFR (EGFR-p) and reversed the inhibition of BBR on NPC proliferation, metastasis, and invasion. Furthermore, BBR-specific SE may trigger autophagy by enhancing EGFR gene transcription, thereby upregulating the RAS–RAF1–MEK1/2–ERK1/2 signaling pathway. In addition, in vivo BBR effectively inhibited NPC cells growth and metastasis, following an increase LC3 and EGFR and a decrease p62. Collectively, this study identifies a novel BBR-special SE and established a new epigenetic paradigm, by which BBR regulates autophagy, inhibits proliferation, metastasis, and invasion. It provides a rationale for BBR application as the treatment regime in NPC therapy in future.

**Supplementary Information:**

The online version contains supplementary material available at 10.1186/s11658-024-00607-4.

## Introduction

Nasopharyngeal carcinoma (NPC) is a prevalent malignant tumor of the head and neck, particularly in the southern region of China [1]. At present, patients with NPC are treated by radiotherapy or chemotherapy [2], but drug resistance, hepatotoxicity, and recurrence after treatment is a big problem in patients with NPC [3–5]. Hence, there is an urgent need to discover novel medications and investigate new mechanisms for the treatment of NPC. The report shows that various active ingredients extracted from Chinese medicine herbs, for instance, gambogic acid, luteolin, ginkgolic acids, curcumin, maackiain, etc., have little toxicity and stable curative effects for NPC [6–10]. Hence, natural small molecule drugs are getting more and more attention from researchers because of their high efficacy and low side effects.

Chinese medicine monomers are the natural small molecule drug with anticancer properties [11–13]. Berberine (BBR) (from *Coptis chinensis* Franch), possesses multiple pharmacological effects, for example, antiheart failure, anticancer, and antimicrobial effects [14]. Studies have shown that BBR inhibits cell growth, metastasis, and invasion of different cancers through induces autophagy and reduces drug resistance [15–18]. BBR has attracted more and more attention because it reduces drug resistance and reveals significant clinical benefits [19]. The precise mechanism by which BBR inhibits the growth and spread of NPC is still not fully understood.

Autophagy plays a critical role in tumor development, characterized by its capacity to generate double-membrane autophagosomes and induce cellular apoptosis [20]. Autophagy is a double-edged sword. In anticancer therapies, induction of autophagy can promote organelle degradation to inhibit tumor growth [21]. Autophagy not only induces cell death but also reduces resistance to anticancer drugs [22]. Natural small molecule drugs are effective in clinical cancer treatment by inducing autophagy of tumor cells, and the autophagy induced by Chinese medicine monomers are expected to both minimizing toxicity [23]. For example, kaempferol induces autophagy in tumor cells and exerts a significant inhibitory effect on non-small cell lung cancer cells (NSCLC) [24]. Saikosaponin A triggers cancer cell death by increasing autophagy through inactivation of the Akt–mechanistic target of rapamycin (mTOR) signaling pathway [25]. Sophflarine A has the ability to induce autophagy-mediated cell death in NSCLC cells by inhibiting the PI3K/AKT/mTOR signaling pathway [26]. Therefore, search for anticancer drugs to induce tumor cells autophagy had been become a hotspot and is regarded as a new strategy for cancers treatment.

In this study, BBR as a Chinese medicine monomer against NPC displayed obvious NPC inhibition in vitro and vivo. Mechanistically, in combination with the results of chromatin immunoprecipitation (CHIP-seq) and RNA sequencing (RNA-seq) analysis, we have confirmed that the BBR-specific super enhancer (SE) is capable of inducing autophagy in NPC cells by promoting the epidermal growth factor receptor (EGFR) and enhancer the activation of downstream pathways. Therefore, this study identifies a novel mechanism of BBR with potential anti-NPC activity.

## Materials and methods

### Reagents and antibodies

BBR was provided by Cayman Chemicals (10006427, Ann Arbor, Michigan) and has a purity of ≥ 95%. The antibodies including anti-EGFR (ab52894), anti-EGFR-p (ab40815), anti-rapidly accelerated fibrosarcoma (RAF1) (ab181115), and anti-RAS type GTPase family (RAS) (ab52939) were procured from Abcam based in Cambridge, UK. The antibodies including antimicrotubule associated protein 1 light chain 3 (anti-LC3) (14600), anti-p62 (18420), antimitogen-activated protein kinase 3 (anti-ERK1/2) (11257), anti-phosphorylated ERK1/2 (28733), antimitogen-activated protein kinase kinase 2 (anti-MEK1/2) (11049), and antiphosphorylated MEK1/2 (28955) were purchased from Proteintech (Wuhan, China).

### Cell lines and cell culture condition

The human NPC cell lines S18 (cat. number MZ-2715) and 5-8F (cat. number MZ-2346), derived from CNE-2 and CNE-1 cell lines, were obtained from the Shanghai cell Bank. The C666-1 cell line was generously provided to our research group by the Central Laboratory of Hunan Cancer Hospital. The NPC cells were cultured in 50 ml RPMI-1640 medium supplemented with 5 ml fetal bovine serum (Gibco) and 500 µl penicillin–streptomycin.

### CCK-8 assay and detection of LDH

The cell counting kit-8 (CCK-8) assay utilized to assess the impact of drugs on cell proliferation inhibition, and the instructions provided by Biosharp (Bs350A; Anhui, China) were strictly followed [27]. The cells were inoculated into 96-well plates. After the cells were attached to the wall, BBR with different concentration gradients was added for treatment, and a blank control hole was set up. After 1 day, incubate the CCK-8 reaction solution for 1 h, and then, the absorbance is determined and recorded. The detection of lactate dehydrogenase (LDH) test kit (C0016) was purchased from Biyuntian Co. (Shanghai, China) and operated in accordance with the specified requirements [28]. In brief, 45 μl of LDH working solution was added to the cells for detection. Following a 30-min incubation, absorbance was measured, and cell mortality was calculated.

### Wound-healing and transwell assays

A wound-healing experiment was performed by marking a scratch wound with the tip of a pipette, and the photographs were taken using the phase-contrast microscope (Olympus, Japan). Transwell experiments were conducted following the methods described in literature [29].

### Colony formation assays

The cells were inoculated in 6-cm cell culture dish, treated with BBR drugs after the cells had attached to the wall, and cultured for 5 days. Fixed staining was performed to facilitate the observation of the number of cell clusters [30].

### Transmission electron microscopy (TEM)

The samples were treated in accordance with the literature description [31]. After discarding the culture medium from the cells, an adequate amount of 2.5% glutaraldehyde was swiftly added for fixation. After 1 h, the cells were transferred to a centrifuge tube using a cell scraper and then centrifuged at 500 rpm for 10 min. The cells were gently resuspended before being transferred to a 1.5-ml EP tube. After standing vertically for 1 h, the supernatant was carefully aspirated. Subsequently, 1 ml of new glutaraldehyde was added along the wall of the tube before storing it in the refrigerator at 4 ℃ for fixed storage.

### Autophagy staining assay kit with monodansylcadaverine (MDC)

The MDC test kit (C3018S, Shanghai, China) was purchased from Biyuntian Biological Co. and employed following established protocols [32]. The culture medium of the cells was aspirated, and then 1 ml of MDC staining solution was added to each well. After 30 min, the MDC dye was blotted, followed by three washes with assay buffer. Finally, the results were captured using a fluorescence microscope.

### Quantitative real-time polymerase chain reaction (qRT–PCR)

Total RNA was extracted from the cells, and the concentration of RNA was determined. Subsequently, a cDNA template was synthesized through reverse transcription. The amplification reaction system was then configured according to different primers, followed by the performance of the amplification reaction [33]. The primers were tested in accordance with the sequence provided in Supplementary data. Transcription data were analyzed by the 2^−△△Ct^ method.

### CRISPR interference

Two active SE regions in NPC cells were targeted and eliminated using clustered regularly interspaced short palindromic repeats (CRISPR)–CRISPR-associated protein 9 (Cas9) [34]. The primers were tested in accordance with the sequence provided in supplementary data.

### ChIP assays

S18 and S18 cells treated with BBR were processed at the Epibiotek Laboratory (Guangzhou Epibiotek Co., China) for ChIP-seq on H3K27ac marks [35].

### RNA-seq assays

The detection of RNA-seq was conducted in accordance with previous reports [36]. The samples with three biological replicates were sequenced at the Epibiotek Laboratory (Guangzhou Epibiotek Co., China) using the Illumina HiSeq2000 platform.

### Western blotting (WB) analysis

The WB assay was carried out in accordance with the findings documented on existing literature [37]. In brief, the denaturation of protein occurred following the extraction of cell sample protein. Subsequently, a protein gel electrophoresis experiment was conducted. The membrane transfer and chemiluminescence were then performed, followed by statistical analysis of the protein bands.

### Lentivirus transfection

Cell transfection were performed as previously reported [38]. The EGFR lentiviral vectors (LV-EGFR) and its negative control virus (LVCON313) were both procured from Genechem Co. (Shanghai, China).

### Animal studies

Nude mice were provided by SJA Company (Changsha, China). The animal experiment protocol was approved by the Ethics Committee of Medical College of the Affiliated Cancer Hospital of Xiangya School of Medicine (KNZY-202210). Subcutaneous xenograft assays were performed using nude mice (female, 4–6 weeks old, SPF). The mice were injected with S18 cells (1 × 10^6^) subcutaneously and then randomly divided into two groups of five mice each. The BBE group was administered a dose of 15 mg/kg/day for 25 consecutive days, while the control group received 10% dimethyl sulfoxide (DMSO). The dimensions of the tumors in the mice were measured at 5-day intervals. After 4 weeks, the mice were euthanized by inhaling 30% vol/min CO_2_ in accordance with ethical guidelines, and the tumors were excised for photographic documentation. S18 cells (2 × 10^6^) were intravenously injected into mice to establish a lung metastatic tumor model of NPC in nude mice. The mice were randomly divided into two groups: a control group and a BBR group (five mice per group). After 30 days, lung metastases tumors in mice were quantified using multimode in vivo animal imaging (AniView100).

### Immunohistochemistry

Immunohistochemical tests were conducted following the procedures outlined in literature [39]. The tumor tissues of nude mice were fixed, followed by paraffin sectioning and dewaxing with xylene and gradient concentration alcohol. An antigen repair was then performed, followed by sealing with serum. Subsequently, primary and secondary antibody incubation took place, along with the addition of a color development agent and redyeing. Dehydration was carried out before final sealing, after which photographs were taken under the microscope.

### Statistical analysis

The statistical analysis employed either an analysis of variance (ANOVA) test or Student’s *t*-test, with a minimum of three biological replicates conducted for each experiment. The Prism software (version 9.0) is utilized for the analysis and visualization of experimental data. A *P* value < 0.05 was considered to be statistically significant.

## Results

### The effect of BBR on NPC cells growth and its noncytotoxic concentration

**Fig. 1:**
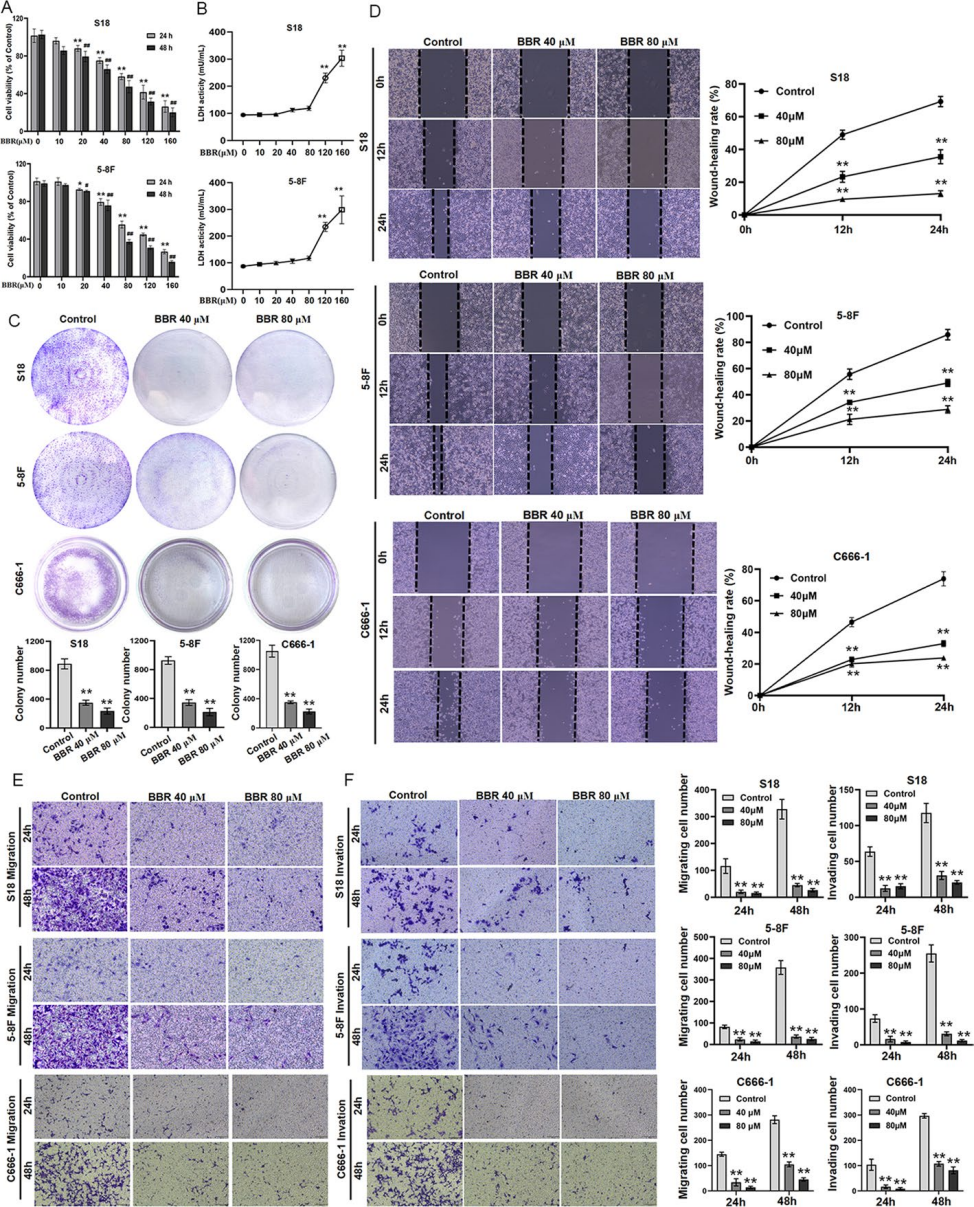
BBR inhibits NPC cell growth and migration. S18 and 5-8F cells were treated with BBR at 10, 20, 40, 80, 120, and 160 μM for 24 or 48 h and subjected to CCK-8 assay for cell viability (**A**). After BBR treatment for 24 h, LDH was detected with LDH assay (**B**) (**P < 0.01 versus BBR 80 μM). Cell proliferation was tested with cell colony growth (**C**). Quantitative analyses of colony numbers are shown (**P < 0.01 versus the control group). A wound-healing assay was used to detect cell migration (**D**). Representative pictures of S18, 5-8F, and C666-1 cells cultured with the different concentrations of BBR in 12 h or 24 h, and the relative migration values were presented as the means ± standard error of the mean (s.e.m.). The Transwell migration and invasion assays were used to detect the migratory and invasive capabilities of S18, 5-8F, and C666-1 cells treated with 40 and 80 μM, and the number of migrated and invaded cells were presented as the means ± s.e.m. (**E**, **F**). All experiments were done more than three times independently and statistically analyzed with one-way analysis of variance (**P < 0.01 versus the control group)

The CCK-8 assay was utilized to evaluate the impact of BBR on the growth of NPC cells. The NPC cells were incubated with BBR of 10–160 μM concentrations for 24 or 48 h, respectively. The 24-h concentration at which a substance exerts half of its maximal inhibitory effect (IC_50_) values of S18 cells and 5–8F cells were 91.84 and 95.02, and BBR significant inhibited the growth of NPC cells in a time- and dose-dependent manner (Fig. 1A). The results revealed a significant increase in LDH values when the BBR concentration more than 80 μM (Fig. 1B). The findings indicated that a BBR ≤ 80 μM was the noncytotoxic concentrations to inhabit the growth of NPC cells, and in subsequent experiments, a BBR of 40 and 80 μM was applied. The colony forming array was utilized to observe the long-term therapeutic effect of BBR on NPC cells. With increasing concentrations of BBR, the colony formation capacity of both cells decreased significantly, as compared with controls (Fig. 1C). The results indicated that BBR significantly suppressed the proliferation of NPC cells. To explore the impact of BBR on metastasis of NPC in vitro, the cells treated with DMSO, 40 μM BBR, and 80 μM BBR were used for the wound-healing assay, migration assay, and invasion assay, respectively. The results demonstrated a significant inhibitory effect of BBR on the migratory and invasive capabilities of NPC cells (Fig. 1D–F).

### BBR induces autophagy in NPC cells

**Fig. 2:**
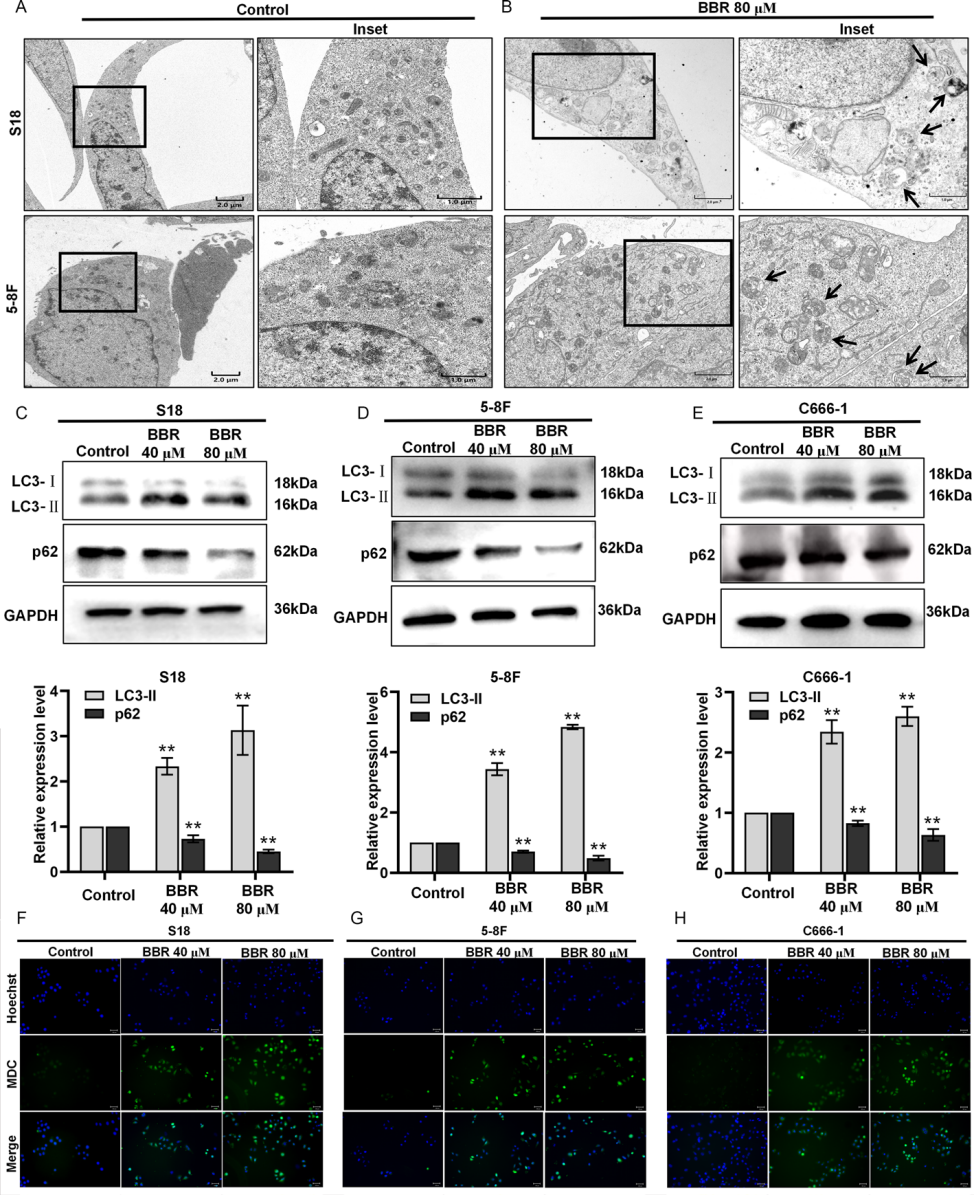
BBR induces autophagy in NPC cells. TEM was used to observe autophagy in S18 and 5-8F cells, and the scale bar represents 2 µM and 1 µM (**A**, **B**). The expressions of LC3 and p62 protein in S18, 5-8F, and C666-1 cells treated with BBR were examined by western blotting (**C**–**E**). MDC-positive staining cells were observed by inverted fluorescence microscope (**F**–**H**). All experiments were done more than three times independently and statistically analyzed with one-way analysis of variance (**P < 0.01 versus the control group)

Autophagy has emerged as a crucial mechanism of tumor cell death triggered by numerous anticancer drugs [40, 41]. TEM is considered the definitive method for assessing autophagy in cells through direct visualization of autophagosome formation, making it the gold standard in research [42, 43]. S18 and 5-8F cells exhibited typical characteristics of autophagy with autophagosomes and autolysosomes after treated with BBR for 24 h (Fig. 2A, B). LC3 is an autophagosomal marker protein [44]. LC3-II protein accumulates during autophagy, and detection of LC3-II has become an effective method to monitor cells autophagy [45]. When autophagy occurs in cells, the levels of the p62 protein decrease significantly [46]. The findings demonstrated an increase in LC3-II protein levels after BBR treatment, and there was a decrease in p62 protein levels, (Fig. 2C–E). The MDC staining technique was employed to examine the alterations in autophagy in NPC cells following treatment with BBR. There was an increase in the number of MDC-positive cells in the BBR treatment group. (Fig. 2E–H). These findings suggest that BBR may effectively induce autophagy in NPC cells.

### Decreased-autophagy attenuates BBR-induced the inhibition of proliferation and metastasis of NPC cells

**Fig. 3:**
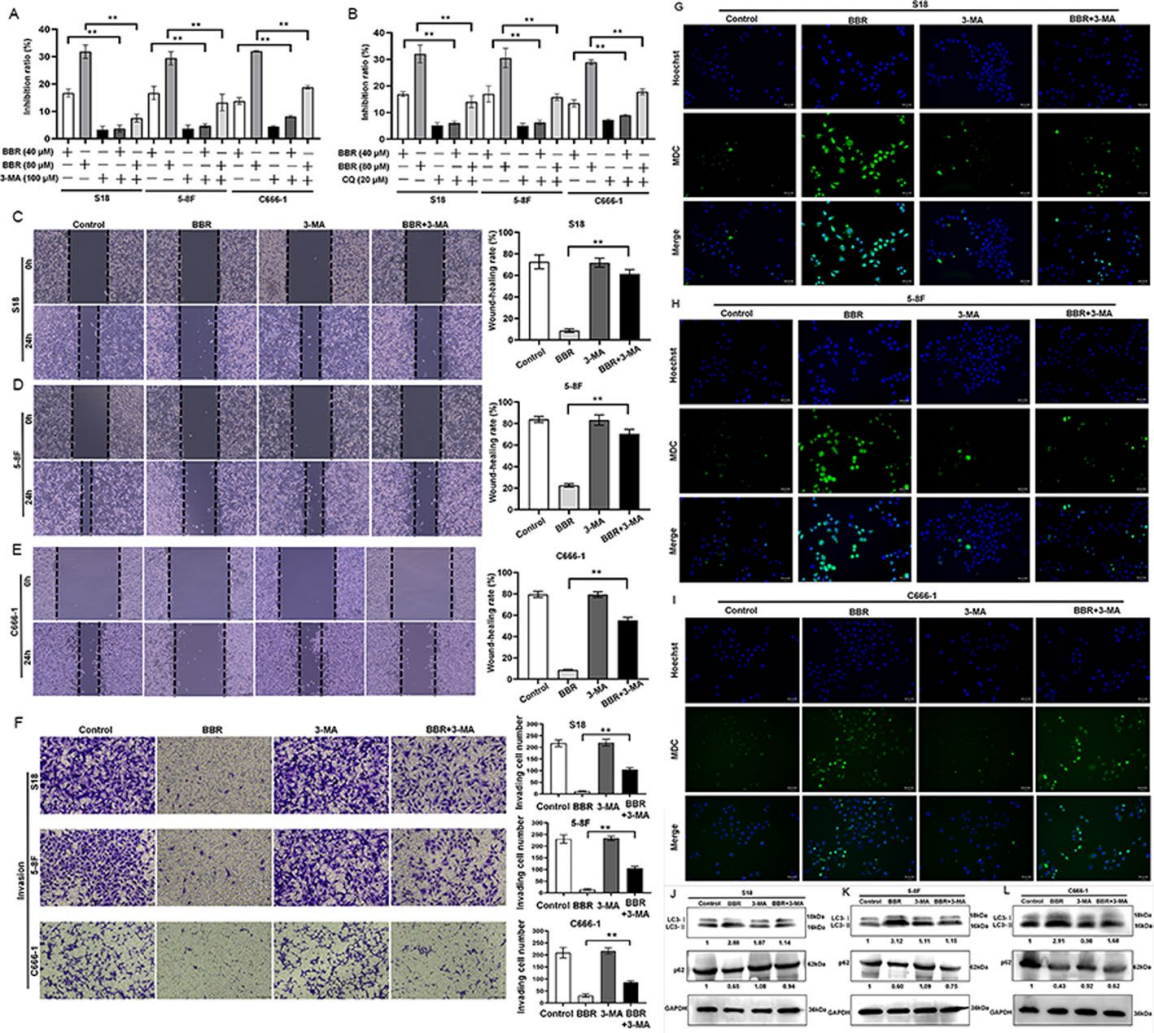
The decreased-autophagy attenuates BBR-induced inhibition on the proliferation and metastasis of NPC cells. S18, 5-8F, and C666-1 cells were treated with BBR with or without CQ, 3-MA for 24 h, and the inhibition of growth was assayed (**A**, **B**) (** P < 0.01, versus the control group). A wound-healing assay was used to detect cell migration. Representative pictures of S18, 5-8F, and C666-1 cells were treated with BBR with or without CQ or 3-MA for 24 h are shown, and the relative migration values were presented as the means ± s.e.m. (**C**–**E**). The transwell invasion assays were used to detect the invasive capabilities of NPC cells. The S18, 5-8F, and C666-1 cells were treated with BBR with or without 3-MA for 24 h, and the number of invaded cells were presented as the means ± s.e.m. (**F**). The MDC-positive staining cells were observed by inverted fluorescence microscope (**G**–**I**). The expressions of LC3 and p62 protein in S18, 5-8F, and C666-1 cells treated with BBR with or without 3-MA for 24 h was examined by western blotting (**J**–**L**). All experiments were done more than three times independently and statistically analyzed with one-way analysis of variance (**P < 0.01 versus the control group)

To investigate the role of the autophagy in BBR-mediated antinasopharyngeal carcinoma effects, we employed autophagy inhibitors [3-methyladenine (3-MA) and chloroquine (CQ)] to block autophagic flux and subsequently evaluated the suppressive efficacy of BBR on NPC cells. The results indicated that treatment with 3-MA or CQ significantly rescued the mortality of BBR-induced NPC cell death. (Fig. 3A, B). Wound-healing assay results showed that treatment with 3-MA significantly rescued the suppression effect of BBR on NPC cell metastasis. (Fig. 3C–E). Transwell assay results demonstrated that treatment with 3-MA attenuated the inhibitory effect of BBR on the invasion of NPC cells (Fig. 3F). Moreover, BBR-induced MDC levels were inhibited by 3-MA, and alone, 3-MA had no influence on MDC staining (Fig. 3G–I). Furthermore, western blotting analysis revealed that 3-MA significantly rescued BBR-induced LC3-II and the inhibition on p62 (Fig. 3J–L). The results implied that BBR induces autophagy could be the main mechanism of its anti-NPC effect.

### BBR induces autophagy by promoting EGFR and its downstream signaling pathway activation

**Fig. 4:**
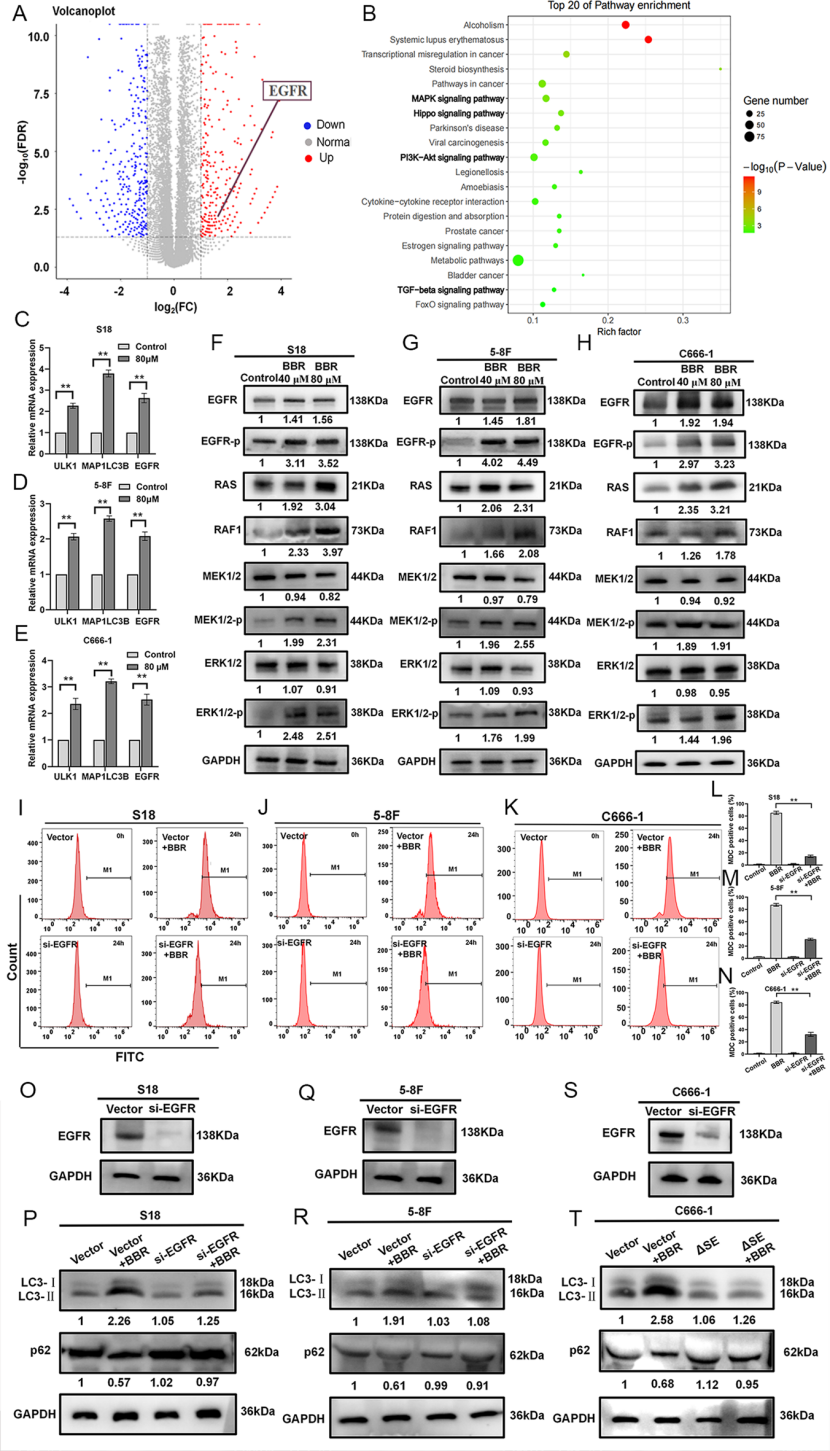
BBR induces autophagy by mediating EGFR and downstream signaling pathway activation. **A** the volcano plots of RNA-seq data (S18-BBR versus S18), FC represents fold change, and FDR represents false discovery rate. **B** Scatter plot of KEGG enrichment of differentially expressed genes in S18 and S18-BBR cells, rich factor represents the number of differential genes located in the KEGG/the total number of genes located in the KEGG. The expression of ULK1, microtubule-associated protein 1 light chain 3 beta (MAP1LC3B), and EGFR mRNA in S18, 5-8F, and C666-1 cells treated with BBR was examined by qRT–PCR. (*n* = 3–5 independent experiments) (**C**–**E**). Data are expressed as mean ± s.e.m.; **P < 0.01. The expressions of EGFR, EGFR-p, RAS, RAF1, MEK1/2, MEK1/2-p, ERK1/2, and ERK1/2-p proteins in S18, 5-8F, and C666-1 cells treated with BBR were examined by western blotting (**F**–**H**). The MDC-positive staining cells level in S18, 5-8F, and C666-1 cells treated with BBR were analyzed by a flow cytometer (**I**–**N**). The expressions of LC3, p62 protein in S18, 5-8F, and C666-1 cells with knockout of EGFR or treated with BBR were examined by western blotting (**O**–**T**)

The molecular mechanism underlying BBR-induced autophagy in NPC cells was elucidated through RNA-seq. The results revealed that, in comparison with the control group, there were 1163 differentially expressed genes in the BBR treatment group (*P* ≤ 0.05, FC ≥ 2) (Fig. 4A). Additionally, the Kyoto Encyclopedia of Genes and Genomes (KEGG) database was employed to ascertain activated pathways in BBR-treated S18 cells, aiming to unveil regulatory factors associated with autophagy. The various autophagy related pathways (TGF-β, PI3K-AKT-mTOR, TNF, and MAPK pathway) were found to be enriched during BBR treatment (Fig. 4B). The mRNA expression levels of autophagy-related genes EGFR, MAPLC3B, and ULK1 were significantly upregulated in the BBR treatment group. The qRT–PCR results demonstrated a significant upregulation in the expression level of EGFR, MAPLC3B, and ULK1 mRNA following BBR treatment (Fig. 4C–E). As EGFR is involved in the MAPK signaling pathway, our results demonstrate that treatment with BBR led to increased levels of EGFR, RAS, RAF1, and phosphorylated EGFR (EGFR-p), MEK1/2 and ERK1/2 (Fig. 4F–H). Hence, following BBR treatment, the MAPK signaling pathway is activated in NPC cells. To further validate whether this finding is related to BBR-induced NPC cell autophagy, the siRNA targeting EGFR was designed to silence EGFR, the knockout of EGFR reversed the BBR-induced increase in LC3-II increase and decrease in p62 in the NPC cells (Fig. 4O–T). Simultaneously, the MDC staining by the flow cytometry assay results also confirmed that knockdown of EGFR reversed BBR-induced MDC increase (Fig. 4I–N), indicating that EGFR plays a pivotal role in BBR-induced autophagy of NPC cells.

### EGFR is driven by a BBR-specific super-enhancer

**Fig. 5:**
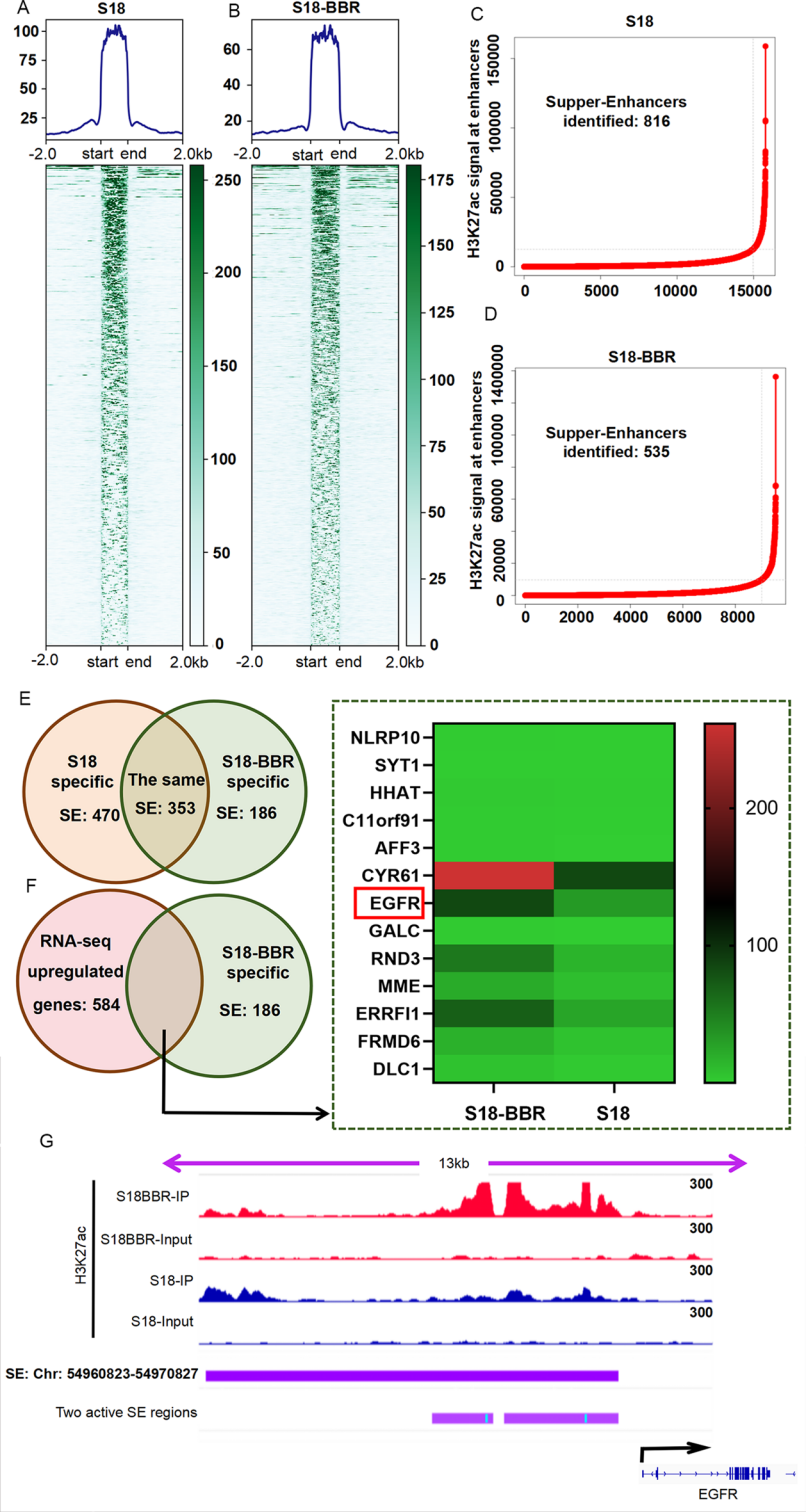
**A**, **B** EGFR is driven by BBR-specific SE. H3K27ac signal in SE regions S18 cells with (**A**) or without BBR (**B**). **C**, **D** The numbers of identified SEs and the H3K27ac signal at enhancers in S18 (**C**) and S18-BBR cells (**D**). **E** the numbers of identical and distinct SEs in S18 and S18-BBR cells were analyzed. **F** Heatmap showed the normalized expression of significantly upregulated genes between S18 and S18-BBR cells. **G** the locations of the S18-BBR specific SE, and the active SE regions were visualized by IGV

**Fig. 6:**
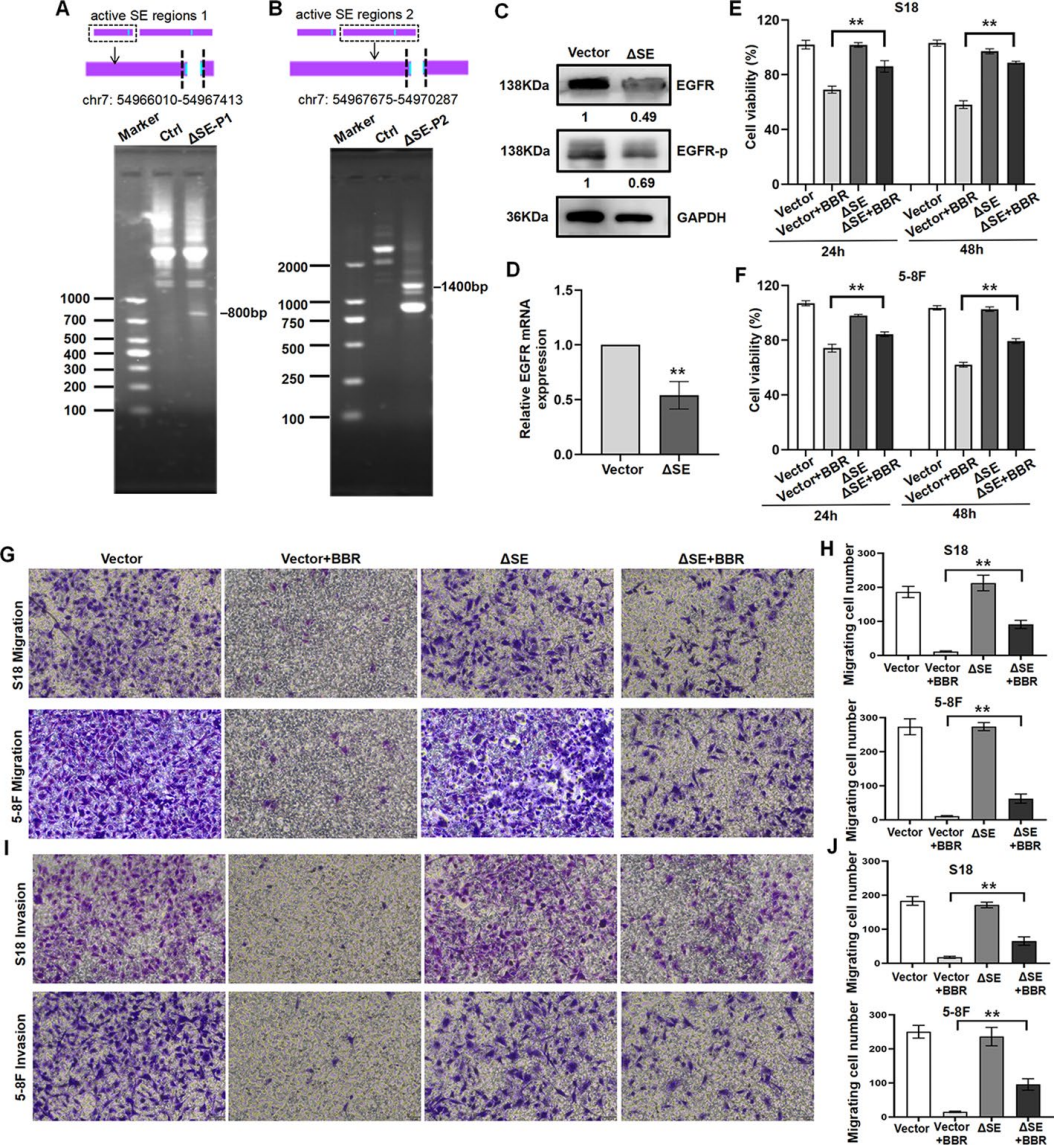
Deletion of BBR-specific SE reverses BBR-induced NPC proliferation and metastasis in vitro. The SE upstream of EGFR promoter was deleted using CRISPR interference, the nested PCR was used to verify the knockout efficiency (**A**, **B**). EGFR mRNA and protein expression in ΔSE S18 cells and vector cells (**C**, **D**). ΔSE S18 cells treated with BBR binding SE deletion (*n* = 3–5 independent experiments). Data are expressed as mean ± s.e.m.; **P < 0.01. Cell viability in ΔSE S18 and 5-8F cells treated with BBR was detected by CCK-8 assay (**E**, **F**), (**P < 0.01 versus vector–BBR group). Transwell migration and invasion assay were used to detect the migratory and invasive capabilities of ΔSE S18 and 5-8F cells treated with BBR, and the number of migrated and invaded cells were presented as the means ± s.e.m. (**G**–**J**) (**P < 0.01 versus vector–BBR group)

SE exerts a robust regulatory influence on the expression of target genes [47]. To understand whether the aberrant transcription of EGFR-mediated BBR was propelled by SEs, ChIP-seq was used to screen the different SEs of S18 cell treated with BBR (S18-BBR) based on the H3K27ac signal strength. The results showed that a total of SEs in S18 and S18-BBR cells were respectively 816 and 535, and 186 of 535 SEs were specifically generated after BBR treatment (Fig. 5A–E). Next, the gene transcriptome and SE landscapes of S18-BBR cells were established by RNA-seq and H3K27ac ChIP-seq. The ROSE algorithm results showed that EGFR may be a SE-driven gene mediated by BBR. Unlike S18 cells, the upstream region of EGFR in S18-BBR cells has a high level of H3K27ac modification, (Fig. 5F, G). The deletion of this SE using CRISPR–Cas9 (Fig. 6A, B) led to decreased the level of EGFR mRNA and protein (Fig. 6C, D). These findings indicate that the SE plays a significant role in BBR’s impact on EGFR transcription and expression in NPC cells.

### Deletion of BBR-specific SE reverses BBR-induced NPC proliferation and metastasis in vitro

To investigate the impact of BBR-specific small molecules on in vitro metastasis and growth of NPC, NPC cells treated with DMSO or BBR and NPC cells with SE deletion treated with DMSO or BBR were used by CCK-8 assay, respectively. These assays demonstrated that the deletion of SE effectively reversed the BBR-induced cell death in NPC cells (Fig. 6E, F). Additionally, the results of the Transwell assay showed that the absence of SE reversed the inhibitory effect of BBR treatment on the migration and invasion of NPC cells (Fig. 6G–J), This suggests that BBR-specific SE could effectively inhibit the migration and invasion of NPC by inducing autophagy.

### BBR inhibits NPC growth and metastasis in vivo

**Fig. 7:**
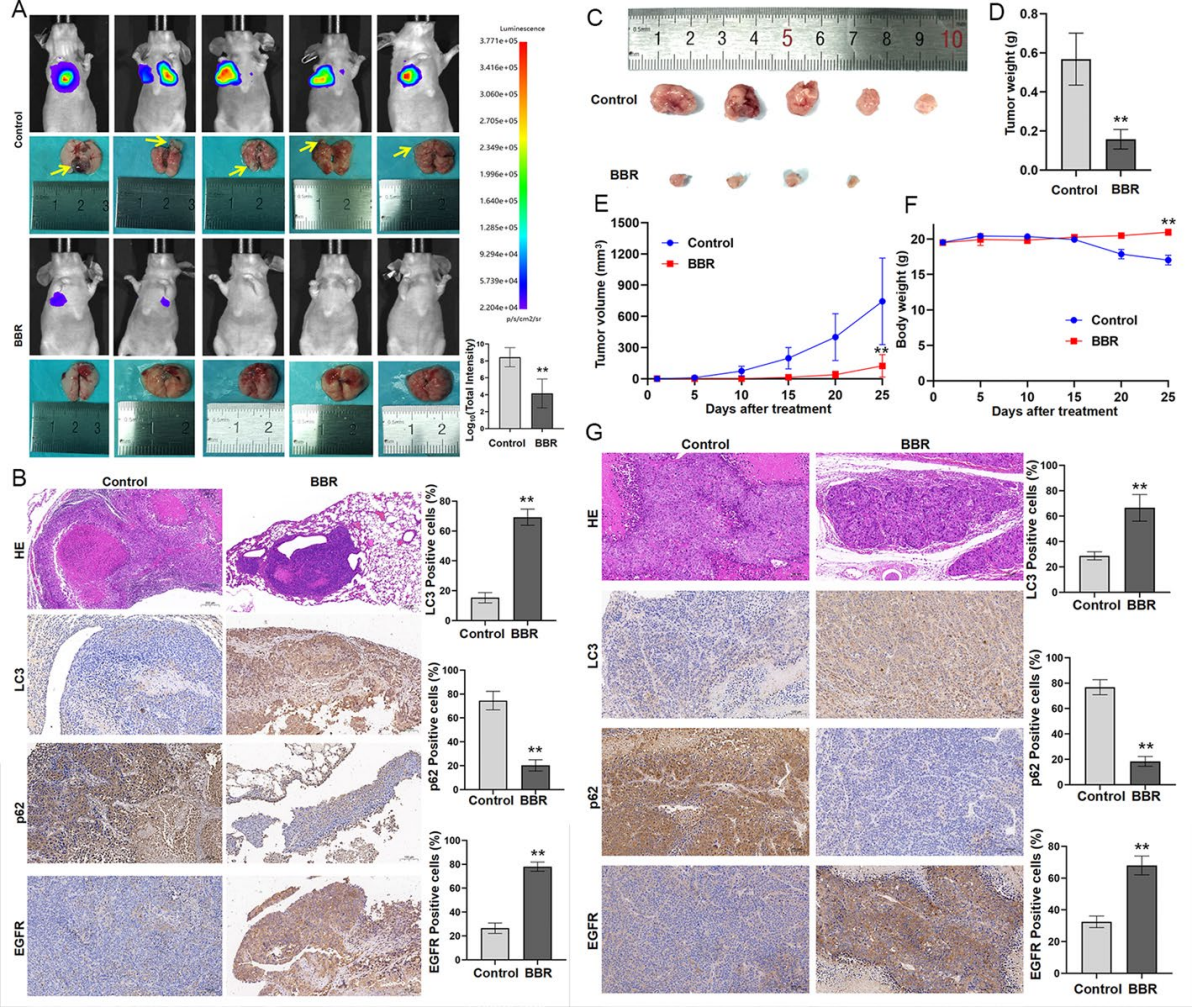
BBR inhibits NPC growth and metastasis in vivo. **A** The S18 cells were injected into the tail vein of nude mice (*n* = 5). The mice were subjected to BBR (15 mg/kg/day) by intraperitoneal injection for 30 day (*n* = 5). The luminescence intensity of lung metastases was analyzed in vivo using an in vivo small animal imaging system (**P < 0.01 versus the control group). **B** Hematoxylin–eosin (HE) staining was used to detect the percent of tumor metastases per lung. The expression of LC3, p62, and EGFR in NPC lung metastatic tissues and NPC primary tissues were measured by IHC staining. **C**,** D** Gross image of subcutaneous tumors and tumor weight in control and BBR group (**P < 0.01 versus the control group). Tumor volume and body weight curve was measured every 5 days, (**P < 0.01 versus day 0) (**E**, **F**). **G** HE staining of the implanted tumors. The expressions of LC3, p62, and EGFR in NPC subcutaneous tumors were measured by IHC staining (**P < 0.01 versus the control group)

The subsequent steps involved investigating the potential of BBR for in vivo metastasis of NPC cells. The mice model of metastatic lung tumors from nasopharyngeal carcinoma was established via tail vein injection, followed by intervention therapy with BBR. The results indicated that, compared with the control group, the BBR treatment group exhibited a reduction in the area of lung metastases in nude mice (Fig. 7 A). Furthermore, the immunohistochemical results demonstrated a concordance between the alterations in autophagy-related protein levels observed in the group treated with BBR and those observed in vitro experiments. Specifically, there was an increase in LC3-II and EGFR protein levels, while p62 protein levels exhibited a decrease (Fig. 7B). To investigate the potential of BBR in inhibiting the proliferation of nasopharyngeal carcinoma in vivo, we subcutaneously injected S18 cells into nude mice and administered BBR drug intervention. BBR treatment could remarkably inhibit NPC growth in vivo (Fig. 7C–F). Immunohistochemical staining revealed an upregulation in LC3-II and EGFR protein levels, accompanied by a notable downregulation of p62 protein levels in the BBR treatment group (Fig. 7G). In conclusion, BBR effectively suppresses the growth and metastasis of nasopharyngeal carcinoma cells both in vivo and in vitro by promoting tumor cell autophagy.

## Discussion

NPC in clinic-alone chemoradiotherapy appears as a severe recurrence and metastasis, so clinicians struggle to find a novel strategy to decrease the recurrence and metastasis of patients with NPC and improve curative effect [48]. In China, the widespread application of Chinese herbal medicine has contributed to survival of patients with tumor including NPC through reducing recurrence. Compared with chemotherapeutic drugs, the use of TCM monomer therapy can result in fewer adverse reactions. BBR is extracted from *Coptis chinensis* and extensively utilized in the treatment of inflammatory and neoplastic diseases [49]. BBR can be used to treat many diseases and has shown beneficial therapeutic effects on pancreatic [50], gastric [51], and ovarian cancer [52].

Research showed that BBR could suppress the growth of NPC cells [53, 54]. This research showed that BBR suppressed NPC growth in a dose-dependent manner and caused remarkable inhibition effect on NPC metastasis in mice xenograft model. These are consistent with the previous reports [53, 54], and BBR has an anti-NPC effect, especially an antimetastasis effect, on NPC.

An accumulating a body of evidence has substantiated the pivotal role of autophagy in pharmacotherapy for NPC [55]. In recent years, it has been discovered that BBR, functioning as an autophagy regulator, exerts inhibitory effects on the proliferation and metastasis of various cancers by inducing autophagy [56–60]. In this study, we demonstrate that BBR induces autophagy in NPC cells in a dose-dependent manner. The study reveals that an elevated level of LC3-II may indicate the occurrence of autophagy [61]. Additionally, it is observed that p62 is degraded during autophagy through its interaction with the LC3-interacting region (LIR) [62]. In the present study, BBR treatment increases LC3-II protein expression and degraded p62 in vitro and in vivo, as well as induces autophagy flux in NPC cells. BBR exerts anti-NPC effect through inducing autophagy.

The question of whether induction of autophagy can inhibit tumor cell growth remains a topic of controversy in the field. Studies have shown that BBR inhibits the growth of tumor cells by inducing autophagy [56]. Conversely, the findings from the reports indicate that inhibition of autophagy enhances BBR-induced cell death, thereby highlighting the cytoprotective role played by autophagy [63]. The inhibitory effects of BBR on growth, metastasis, and invasion were partially reversed by autophagy-specific inhibitors in the present study, thereby confirming the anticancer effect of BBR-induced autophagy. These findings suggest that BBR has the potential to inhibit the proliferation and metastasis of nasopharyngeal carcinoma by inducing autophagy.

To elucidate the molecular mechanisms underlying berberine (BBR)-induced autophagy in NPC cells, we utilized RNA-seq to interrogate the transcriptome of cells treated with BBR, revealing a significant upregulation of EGFR mRNA expression. At the same time, we observed that EGFR downstream molecules RAS–RAF1–MEK1/2–ERK1/2 were aberrant activated. EGFR regulates cellular growth, proliferation, and differentiation [64]. EGFR signaling can regulate tumor cell growth, and targeting the EGFR pathway for anticancer drugs is considered a promising strategy. Significantly, the epidermal growth factor receptor (EGFR) or its downstream signaling pathways have been shown to play a crucial role in determining whether autophagy results in cytoprotective or cytotoxic effects [65–67]. Xu Wang et al. revealed that PTBP1-mediated enhancement of breast cancer cell proliferation and invasiveness is facilitated through autophagy induction via the PI3K/Akt signaling pathway [68]. The autophagic apoptosis in NPC cells was found to be induced by sodium butyrate (NaBu) through the inhibition of the AKT/mTOR pathway, as reported by Huang et al. [69]. The bidirectional impact of autophagy on EGFR-targeted tumor therapeutic responses underscores the intricate and uncertain nature of cancer treatment. The EGFR-mediated RAS/ERK signaling pathway has the potential to impact cell growth or apoptosis [70]. At present, our results show that RAF1-mediated MEK/ERK activation increased LC3 levels, which is enough to trigger an variation of autophagic flux, indicating the occurrence of autophagy [71]. Pinghu Zhang et al. reported that w09, a novel autophagy enhancer, activated the EGFR-mediated RAS–RAF1–MEK1/2–ERK1/2 signaling cascade to induce autophagy in gastric cancer cells. Jinhua Chen et al. demonstrated that chaetoglobosin G inhibited NSCLC cell proliferation by inducing autophagy through EGFR/MEK/ERK/LC3 pathway to exert an antitumor role. In our study, the EGFR, EGFR-p, RAS, RAF1, MEK1/2, and ERK1/2 proteins were elevated in BBR treatment groups. BBR-induced NPC cell autophagy was partly reversed by EGFR gene knockout. Therefore, EGFR may be used as a pivotal target of BBR-induced autophagy in NPC cells. Subsequently, we investigated the underlying mechanism by which BBR induces an elevation in EGFR levels in NPC cells.

SEs play a pivotal role in orchestrating cell-type-specific gene expression through intricate interactions with master transcription factors (TFs), cofactors, RNA polymerase II, and noncoding RNAs [72]. In this work, BBR-specific SE was identified using H3K27ac ChIP-seq, and knockout SE by CRISPR–Cas9 resulted in downregulation of EGFR and EGFR-p. Reversal of the inhibition of proliferation, metastasis, and invasion mediated by BBR treatment in NPC cells was observed upon knockout of SE. When BBR is present at a low concentration (1 μM), it initially accumulates in the cell mitochondria and plays a role. However, when BBR is in high concentrations (10–100 μM), it will accumulate in the nucleus and play a role [73]. However, SE-driven cell-type-specific genes can form transcriptional condensates in the nucleus [74], and the small-molecule drugs (such as cisplatin) may concentrate in certain condensates and modifies SE DNA [75]. Therefore, a small molecule drug bonding with concentrate preferentially may contribute to enhancement of the pharmacological properties of these drugs. Therefore, we were supposed that BBR may bond with SE concentrate in NPC cell nucleus and promoted EGFR transcription.

## Conclusion

**Fig. 8:**
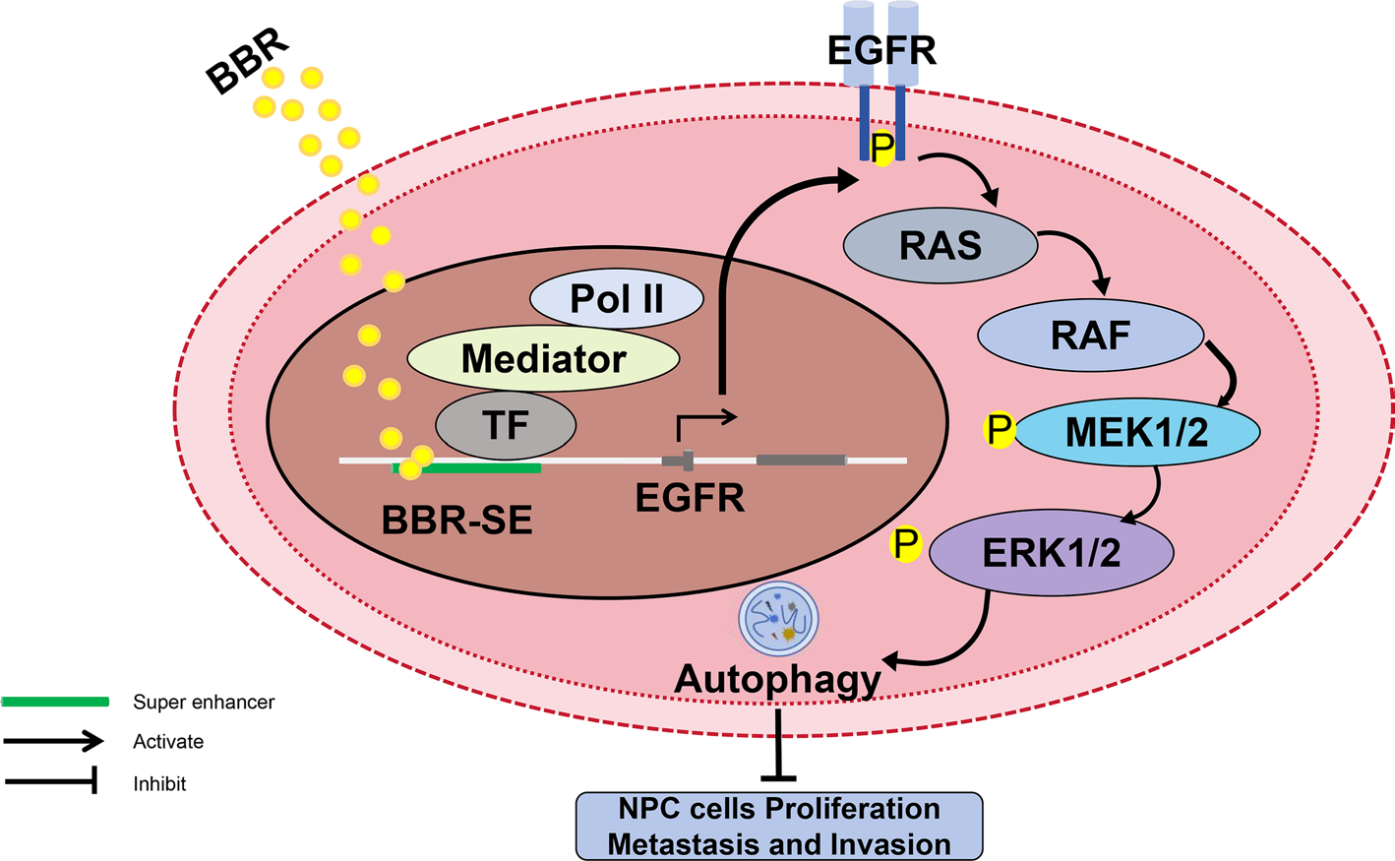
Schematic diagram of berberine-actuated SE regulating EGFR to induce NPC autophagy

This study indicated that BBR exerts inhibitory effects on the growth and metastasis of NPC by inducing autophagy, and this autophagy is regulated by BBR-specific SE. BBR-specific SE regulates the EGFR-mediated RAS–RAF1–MEK1/2–ERK1/2 pathway to induce NPC cell autophagy (Fig. 8). These findings provide novel evidence supporting the potential efficacy of BBR as a promising therapeutic agent for NPC.

### Supplementary Information

Supplementary Material 1.

Supplementary Material 2.

Supplementary Material 3.

## Data Availability

All data generated or analyzed during this study are included in this published article.

## Abbreviations

NPC: Nasopharyngeal carcinoma
BBR: Berberine
NSCLC: Non-small cell lung cancer cells
SE: Super enhancer
CCK-8: Cell counting kit-8
LDH: Detection of lactate dehydrogenase
TEM: Transmission electron microscopy
MDC: Monodansylcadaverine
qRT–PCR: Quantitative real-time polymerase chain reaction
WB: Western blotting
SQSTM1: Sequestosome 1
3-MA: 3-Methyladenine
CQ: Chloroquine
KEGG: Kyoto Encyclopedia of Genes and Genomes
LIR: LC3-interacting region
RNA-seq: RNA sequencing
EGFR: Epidermal growth factor receptor
NaBu: Sodium butyrate
TFs: Transcription factors
DMSO: Dimethyl sulfoxide
LC3: Microtubule associated protein 1 light chain 3
MAP1LC3B: Microtubule-associated protein 1 light chain 3 beta
MEK1/2: Mitogen-activated protein kinase kinase 2
ERK1/2: Mitogen-activated protein kinase 3
MTOR: Mechanistic target of rapamycin
RAS: RAS type GTPase family
RAF1: Rapidly accelerated fibrosarcoma
