# Supplementary material for: A specific super-enhancer actuated by berberine regulates EGFR-mediated RAS–RAF1–MEK1/2–ERK1/2 pathway to induce nasopharyngeal carcinoma autophagy
Source: Cell Mol Biol Lett. 2024 Jun 28;29:92. doi: 10.1186/s11658-024-00607-4 (PMC11214260; doi:10.1186/s11658-024-00607-4)
Supplement: Supplementary file 1 — Supplementary Material 1. [file 11658_2024_607_MOESM1_ESM.pdf]

Result of STR Detection

1. Basic information

Polyallelic	Cell line to be tested	Cell bank	EV value	Match description
Yes	C666-1	EXPASY	0.92	Almost match

质检专用章

- Polyallelic refers to the phenomenon of genes with three or more alleles.
- The results of cell typing in this test were good.

2. Description of samples

- cell line was found **basically matching** the DNA typing of this cell line incell line search, which is named **C666-1** in the EXPASY database. The cell number corresponded to **CVCL_7949**. In this test, **multiple alleles were found** in this cell line.

EV	Cell No.	Cell name	Locus names								
			D5S818	D13S317	D7S820	D16S539	VWA	TH01	AM	TPOX	CSF1PO
	Query (Your Cell)		11,11,	8,11,	11,12,	10,10,	17,18,	6,8,	X,Y,	8,10,11	11,15,16
0.92(33/36)	CVCL_7949	C666-1	['11', '12']	['8', '11']	['11', '12']	['10', '10']	['17', '18']	['6', '8']	['X', 'Y']	['8', '11']	['11', '15', '16']

Note: The cell lines to be tested are compared with the STR data of cell lines included in ATCC, DSMZ, JCRB and RIKEN databases. Cell lines that are not included in the above cell libraries will not be matched.

3. Typing results of samples

Genotyping results of STR loci and Amelogenin loci in cells						
Loci	STR information of the tested cell			STR information of the cell in cell bank		
	Name of the tested cell: C666-1			Name of the cell in cell bank: C666-1		
	Allele1	Allele2	Allele3	Allele1	Allele2	Allele3
D5S818	11	11		11	12	
D13S317	8	11		8	11	
D7S820	11	12		11	12	
D16S539	10	10		10	10	
VWA	17	18		17	18	
TH01	6	8		6	8	
AMEL	X	Y		X	Y	
TPOX	8	10	11	8	11	
CSF1PO	11	15	16	11	15	16
D12S391	23	23				
FGA	24	24				
D2S1338	16	22				
D21S11	28	31.2				
D18S51	15	16				
D8S1179	11	16	17			
D3S1358	16	17				
D6S1043	18	19				
PENTAE	11	15				

D19S433	12	15.2				
PENTAD	9	10				
D1S1656	13	17.3				

1. STR database comparison

Our company uses DSMZ tools for cell line comparison, which contains 2455 cell line STR data from ATCC, DSMZ, JCRB and RIKEN databases. If the cells to be tested are not included in the above cell libraries or are self-established new cell lines, comparison will not be possible. Users need to compare the cell typing results with other databases.

长沙艾碧维生物科技有限公司
质检专用章

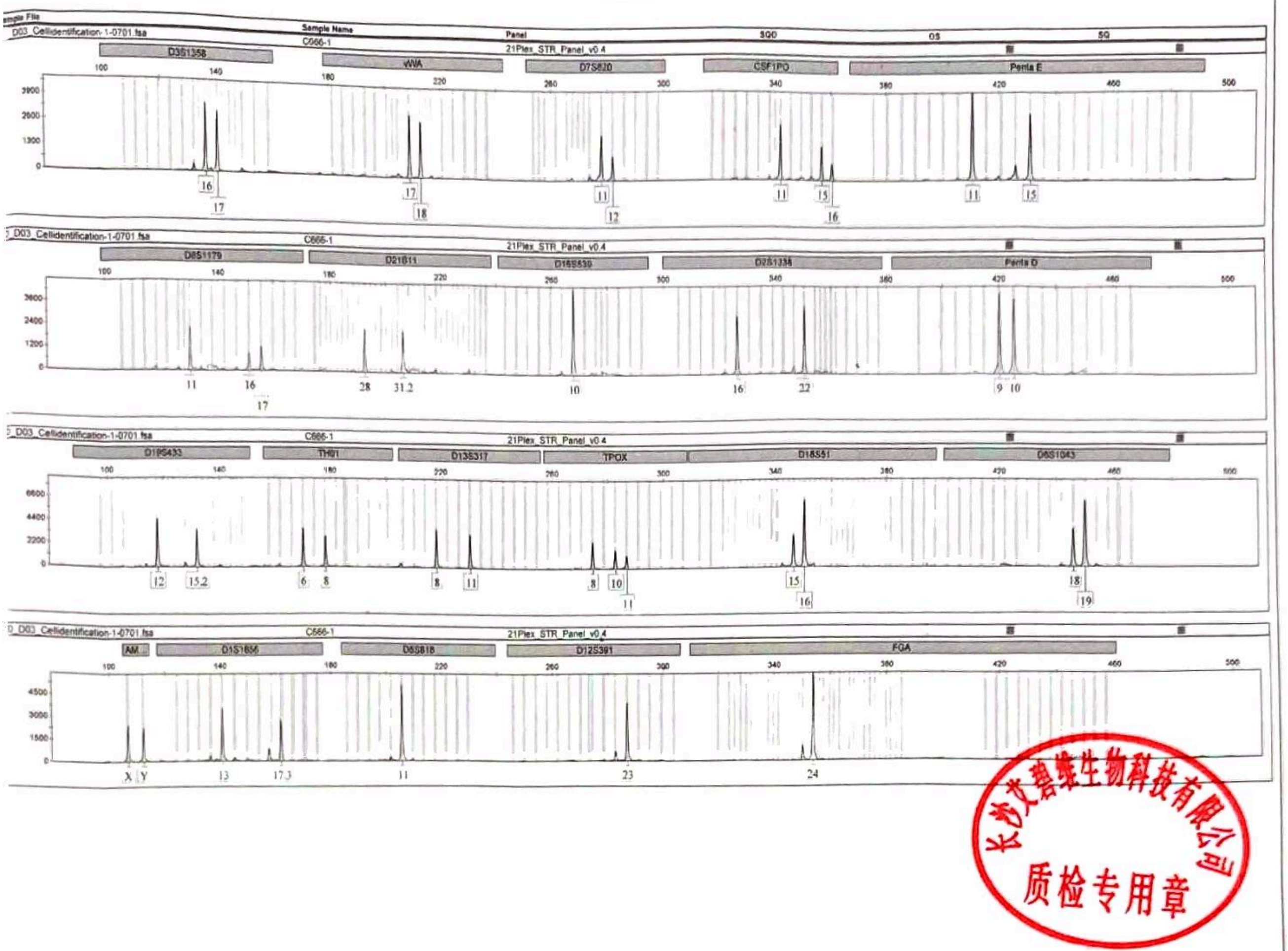

长沙艾碧维生物科技有限公司
质检专用章

CERTIFICATION OF ANALYSIS

Name:SUNE-1 5-8F

Growth Properties:Adherent

Volume/Ampule: T25

Transport Conditions: Room-temperature

Storage Conditions: Liquid nitrogen

Quality Control:

Test Items	Specification	Result
Viability	Trypan Blue Staining	>90%
Doubing time	MTT Testing	48-72 hour
Bacteria/ fungus contamination	Microscopic examination	None detected
Mycoplasma contamination	PCR detection	None detected

Note：This product is for research use only

PCR for mycoplasma detection

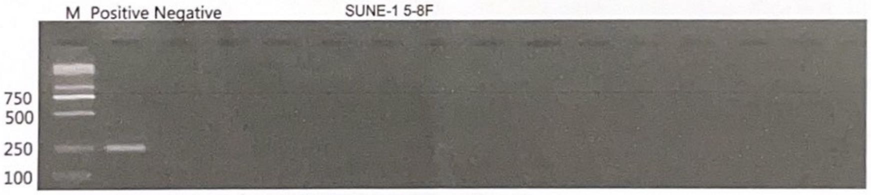

Certified by ： [illegible]

Date：2020.6.17

CERTIFICATION OF ANALYSIS

Name:6-10B

Growth Properties:Adherent

Volume/Ampule: T25

Transport Conditions: Room-temperature

Storage Conditions: Liquid nitrogen

Quality Control:

Test Items	Specification	Result
Viability	Trypan Blue Staining	>90%
Doubing time	MTT Testing	48-72 hour
Bacteria/ fungus contamination	Microscopic examination	None detected
Mycoplasma contamination	PCR detection	None detected

Note：This product is for research use only

PCR for mycoplasma detection

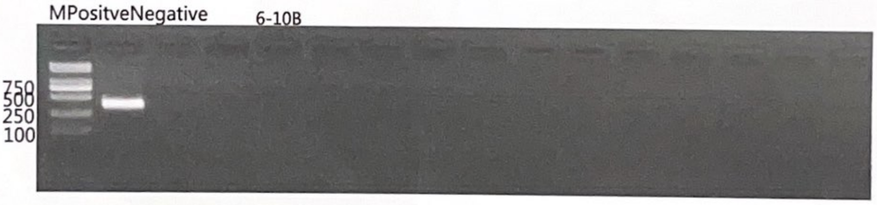

Certified by ： 涂继刚

Date：2020.1.7

Cell STR Certification Report

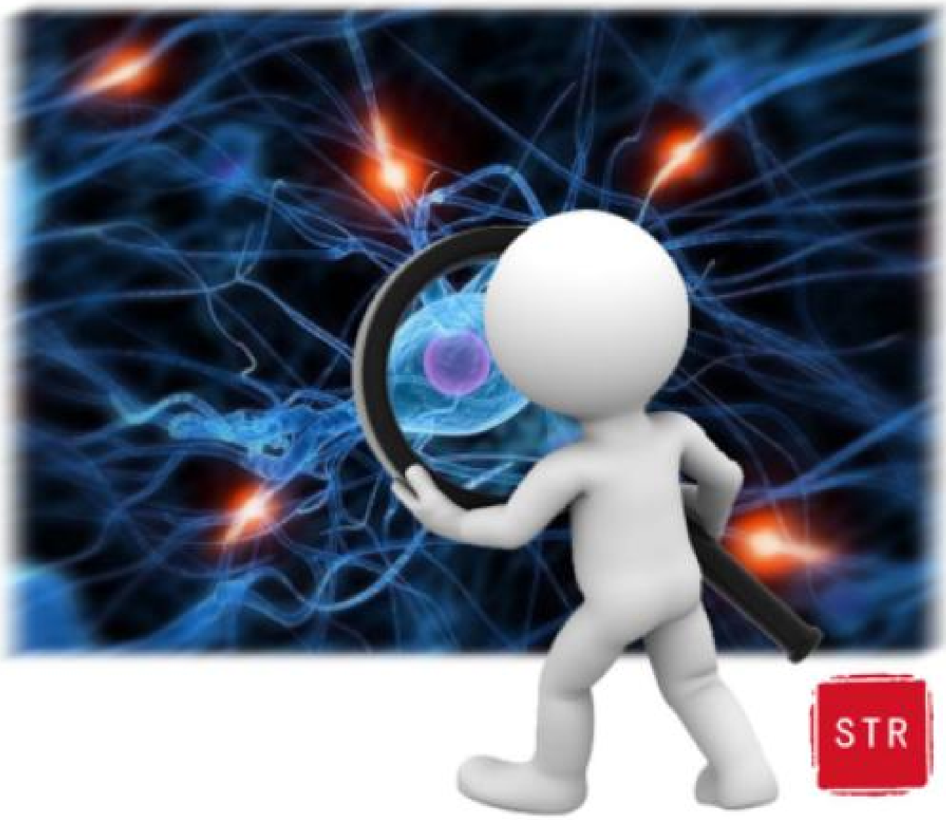

1、 Sample ID：S18

2、 Original Material：Cell pellets

3、 Check time:2019-7-22

4、 Methods：

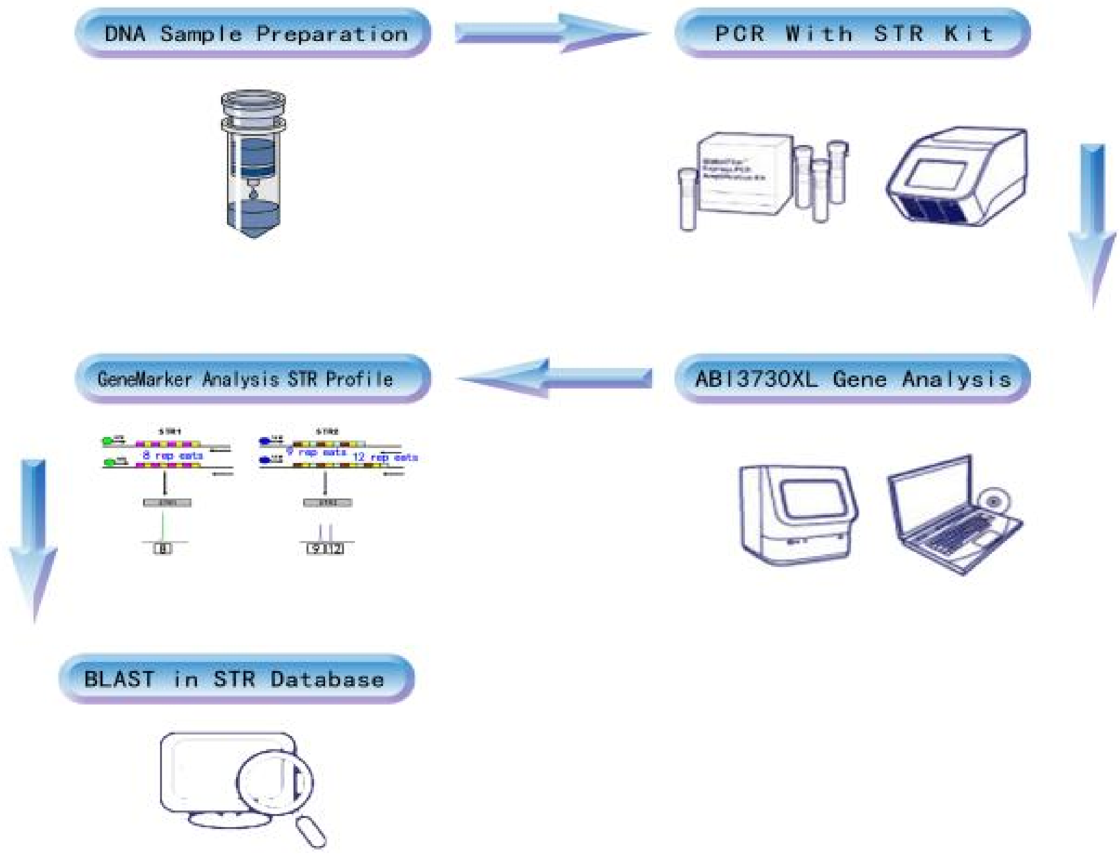

5、 Results：

Negative and positive test results are correct.

Amplification map of Genomic DNA clear，Genotyping results well.

STR Profile :

<table>
<tr><td>Genetic Site</td><td colspan="3">Customer sample</td><td colspan="2">ATCC</td></tr>
<tr><td></td><td colspan="3">S18</td><td colspan="2"></td></tr>
<tr><td>Amelogenin</td><td colspan="2">X</td><td>X</td><td></td><td></td></tr>
<tr><td>CSF1PO</td><td colspan="2">10</td><td>11</td><td></td><td></td></tr>
<tr><td>D13S317</td><td colspan="2">10</td><td>12</td><td></td><td></td></tr>
<tr><td>D16S539</td><td colspan="2">9</td><td>10</td><td></td><td></td></tr>
<tr><td>D5S818</td><td colspan="2">12</td><td>12</td><td></td><td></td></tr>
<tr><td>D7S820</td><td colspan="2">10</td><td>10</td><td></td><td></td></tr>
<tr><td>THO1</td><td>6</td><td>7</td><td>9</td><td></td><td></td></tr>
<tr><td>TPOX</td><td colspan="2">8</td><td>9</td><td></td><td></td></tr>
<tr><td>vWA</td><td colspan="2">14</td><td>14</td><td></td><td></td></tr>
<tr><td colspan="6">Percent match between the sample and the database profile：</td></tr>
</table>

6、 Summary：

The result of STR profile showed no more than 2 distinct alleles were found ,the sample derived from a common ancestry(Figure 1); There are no match cell lines in these Databases of ATCC, DSMZ, JCRB and RIKEN(Figure 2).

Notes：

- P=100% x (2xM)/N; M: number of the matching peaks; N: number of all peaks
  For example:M=5, N=36,P=100% x (2x5)/36=27.7%
- Based on ASN-0002-2011 Standard， cell lines with ≥80% match are considered to be related ; i.e.,derived from a common ancestry. Cell lines with between a 55% to 80% match require futher profiling for authentication of relatedness.
- This data and analysis are for research use only.

Operator: Yuqiu Qin

Auditor: Jigang Tu

GUANGZHOU CELLBANK BIOTECH CO.,LTD
CELL STR PROFILING
PASS

Report time: 2019-7-25

Figure：

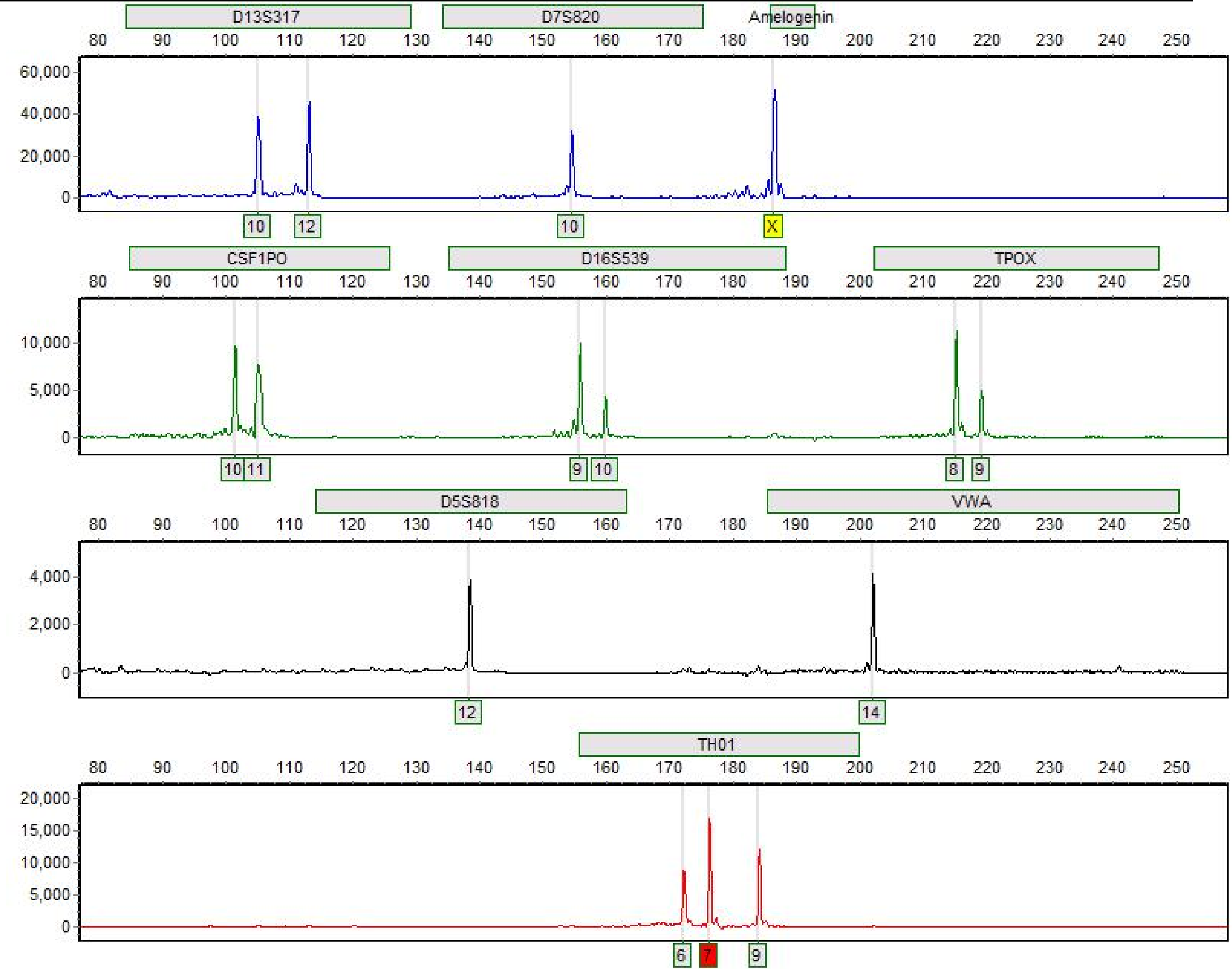

Figure 1.STR profiles of S18 cell line

Result of STR matching analysis by your data.

- DSMZ Profile Database -

A graphical presentation is shown at the bottom of this page.

EV	Cell No.	Cell name	Locus names									Figures
			D5S818	D13S317	D7S820	D16S539	VWA	TH01	AM	TPOX	CSF1PO	
	Query (Your Cell)		*12,12*	*10,12*	*10,10*	*9,10*	*14,14*	*6,7,9*	*x,x*	*8,9*	*10,11*	
0.70(26/37)	RCB1186	TGBC14TKB	12,12	12,12	10,10	9,9	14,14	6,7	X,X	8,13	12,12	-
0.65(24/37)	580	HTC-C3	11,12	10,12	11,12	9,12	14,15	6,7	X,X	8,9	11,12	-
0.65(24/37)	CRL-1366	Or De	11,12	11,12	10,10	9,12	14,17	6,7	X,X	8,8	10,10	-
0.65(24/37)	CRL-2098	SJSA-1	12,12	12,13	9,11	9,10	19,19	7,9	X,Y	8,9	10,11	-
0.65(24/37)	JCRB0164	HTC/C3	11,12	10,12	11,12	9,12	14,15	6,7	X,X	8,9	11,12	-
0.65(24/37)	RCB0452	HTC/C3	11,12	10,12	11,12	9,12	14,15	6,7	X,X	8,9	11,12	-
0.65(24/37)	RCB0520	WR216	10,13	12,14	8,12	9,12	14,14	6,9	X,X	8,9	10,11	-
0.65(24/37)	RCB0587	SF8759	11,12	11,12	10,11	9,9	14,18	6,9	X,X	8,9	10,12	-
0.63(24/38)	JCRB1195	KMS-34	12,12	8,8	10,11	10,13,14	14,14	7,9	X,X	8,9	12,12	-
0.62(26/42)	CRL-4026	THEECs	11,12,13	8,10,12	9,10,11	9,12	14,15,18	7,8,9	X,X	8,10	10,11	-

Figure 2.**compare the result in** DSMZ Database
