# Supplementary material for: A specific super-enhancer actuated by berberine regulates EGFR-mediated RAS–RAF1–MEK1/2–ERK1/2 pathway to induce nasopharyngeal carcinoma autophagy
Source: Cell Mol Biol Lett. 2024 Jun 28;29:92. doi: 10.1186/s11658-024-00607-4 (PMC11214260; doi:10.1186/s11658-024-00607-4)
Supplement: Supplementary file 2 — Supplementary Material 2. [file 11658_2024_607_MOESM2_ESM.pdf]

HPV detection in S18 and 5-8F cells

To verify whether NPC cell lines, S18 and 5-8F are contaminated by Hela cells and display HPV-18 positive (mentioned by the reviewer), HPV was detected in S18, 5-8F and C666-1 cells using HPV nucleic acid detection Kit (No: 20173404697. Tellgen, Shanghai, China). Briefly, the experiments of HPV detection were performed according to the instructions of Human papillomavirus nucleic acid typing detection Kit. Firstly, multiple PCR amplification of sample DNA was performed by primers, and the DNA of 27 kinds of HPV was detected by flow fluorescence hybridization typing. The test results showed that all subtypes of HPV including HPV-18 are negative in S18, 5-8F and C666-1 cells (shown in the Supplementary Data 2).

Microsoft Excel - Output

文件(F) 编辑(E) 视图(V) 插入(I) 格式(O) 工具(T) 数据(D) 窗口(W) 帮助(H)

宋体 12

G87 HPV18

	A	B	C	D	E	F	G	H	I	J	K	L	M
49	32	<unnamed>	2215	10.5	18	13	1062	12	17	12	19.5	12	
50	33	<unnamed>	1831.5	20	25	1407.5	13.5	20	10.5	13	14	16.5	
51	34	<unnamed>	2711	17	9.5	1467.5	23	21	9.5	19.5	6.5	6	
52	35	<unnamed>	2815	8	19	25.5	14	14.5	13	10	18	10	
53	36	<unnamed>	2340	2	9	11.5	22.5	13	14	11	15	14	
54	37	<unnamed>	3311.5	22.5	15	12	15.5	16	23	23	13	13.5	
55	38	<unnamed>	2364.5	25	12	14.5	27	9	20	19	20	19.5	
56	39	<unnamed>	1739	9.5	15.5	8	24	20	19	20.5	14	19	
57	40	<unnamed>	2291.5	12	15.5	18	21	8.5	920	2	12	19	
58	41	<unnamed>	2602	12.5	14	16	19.5	19	26.5	10	18	11	
59	42	<unnamed>	2146.5	19	12.5	13	21.5	27	7	15	15	0.5	
60	43	<unnamed>	2137.5	16	24	1418	25	20	14.5	13	17	19	
61	44	<unnamed>	2515.5	30.5	16	20.5	20	17	23.5	17	11	12	
62	45	<unnamed>	2828	13.5	15	14.5	15.5	16.5	7	20	8.5	23	
63	46	<unnamed>	1637	12	11	21	7	22.5	16	18	16.5	17	
64	47	<unnamed>	2957	17	12	13.5	18	8.5	3	20	17	14	
65	48	<unnamed>	1792	17	14	19.5	17	14	12	8	16	3.5	
66	49	<unnamed>	1843	18	18	22.5	34	15	12.5	11	17	17	
67	50	<unnamed>	3637.5	18.5	22.5	16.5	29	18	10.5	15	15	20	
68	51	<unnamed>	1955	16	30	5.5	30.5	19	16.5	19	15	26	
69	52	<unnamed>	2069	23	11.5	1866	26	15	14	13.5	19	19.5	
70	53	<unnamed>	1858	13.5	26	17.5	28	19	22	18.5	10	17	
71	54	<unnamed>	2204	23.5	15.5	32.5	26	18	18	2	14	21	
72	55	<unnamed>	2711	12	11	25.5	26	12	16.5	15.5	0	13	
73	56	<unnamed>	1877	7.5	18.5	29.5	27	26	3	12.5	1.5	18	
74	57	<unnamed>	2018.5	10.5	26	14	21	14.5	11	15	16	10	
75	58	<unnamed>	2918.5	13	27	20.5	22.5	15	18	17	6.5	13	
76	59	<unnamed>	2690	18.5	12.5	19.5	33.5	18.5	8	12.5	14	12	
77	60	<unnamed>	3916	3	5	9	16	12	8	20	15	16	
78	61	<unnamed>	2214.5	3	16.5	6.5	31	13.5	0	9	17	13.5	
79	62	<unnamed>	2642	10	20	26	30.5	14	10	30	16	15	
80	63	<unnamed>	2045.5	16	16	13	35	15	14	11	14	7.5	
81	64	<unnamed>	[illegible]	11	13	20	41	18.5	13	27	17	18	
82	65	<C666>	25	27	17	17.5	35.5	17	7	18.5	19	5.5	
83	66	<s18>	29	20.5	18	18	26	24	18	9	14	15.5	
84	67	<5-8F>	20	13	12.5	18	30	15.5	19	10	24	3	
85	68	<HPV>	3070	36	27	2728	2448	19	15	12	14	15	
86	69	<Negtive	23.5	17	12	0	32	13	22	17	9	13	
87	Location	Sample	Globin	HPV06	HPV11	HPV16	HPV18	HPV26	HPV31	HPV33	HPV35	HPV39	HPV[illegible]
88	DataType:	Result											
89	Location	Sample	Globin	HPV06	HPV11	HPV16	HPV18	HPV26	HPV31	HPV33	HPV35	HPV39	HPV[illegible]
90	1	<unnamed>	(106026)										
91	2	<unnamed>	(106027)										
92	3	<unnamed>	(106028)										

Output

就绪

开始 hpv, 2024-4-29, 14... Microsoft Excel ...
