# Supplementary material for: A specific super-enhancer actuated by berberine regulates EGFR-mediated RAS–RAF1–MEK1/2–ERK1/2 pathway to induce nasopharyngeal carcinoma autophagy
Source: Cell Mol Biol Lett. 2024 Jun 28;29:92. doi: 10.1186/s11658-024-00607-4 (PMC11214260; doi:10.1186/s11658-024-00607-4)
Supplement: Supplementary file 3 — Supplementary Material 3. [file 11658_2024_607_MOESM3_ESM.docx]

**Supplementary Data 1. The primers sequences for qRT-PCR used in this study**

| Primer | Sequence（5' to 3'） | Product size |
| --- | --- | --- |
| EGFR F | GTGTGCCACCTGTGCCATCC | 20 |
| EGFR R | GCCACCACCAGCAGCAAGAG | 20 |
| MAPLC3B F | GCCTTCTTCCTCCTGGTGAATGG | 23 |
| MAPLC3B R | ATTGCTGTCCCGAATGTCTCCTG | 23 |
| ULK1 F | GGACGCTGCCCGACCTCTC | 19 |
| ULK1 R | GGCGGCTGGTGCTGAAAGAC | 20 |
| GAPDH F | TGACATCAAGAAGGTGGTGAAGCAG | 25 |
| GAPDH R | GTGTCGCTGTTGAAGTCAGAGGAG | 24 |
| Target 1 Peripheral primers F | TGAGGCCAGAATAACTTAGATACCAAG | 27 |
| Target 1 Peripheral primers R | CTGTATAAGCTTCTGCTAGATTCTTCTGT | 29 |
| Target 2 Peripheral primers F | CATGTGGTGAAATTCAAATACGACCA | 26 |
| Target 2 Peripheral primers R | GAGAATGCCCACAGGATATCAAG | 23 |
| Target 3 Peripheral primers F | TGTCTGGACCATAACAGCAGTTG | 23 |
| Target 3 Peripheral primers R | GTTGTGCATCTGCCTTGATCAAG | 23 |
| Target 4 Peripheral primers F | GTAAGTGAAGGTTTATGTGCTGC | 23 |
| Target 4 Peripheral primers R | CTCTGATCTCCCGTACCTATGAA | 23 |
